# Enthesopathy of the bicipital tuberosity of the radius treated under intraoperative computed tomography

**DOI:** 10.1186/s40001-022-00659-2

**Published:** 2022-03-03

**Authors:** Ryosuke Ikeguchi, Takashi Noguchi, Maki Ando, Koichi Yoshimoto, Daichi Sakamoto, Shuichi Matsuda

**Affiliations:** Department of Orthopaedic Surgery, Kyoto University Graduate School of Medicine, 53 Kawahara-Cho, Shogoin, Sakyo-Ku, Kyoto, 606-8507 Japan

**Keywords:** Enthesopathy, Bicipital tuberosity, Radius, Computed tomography

## Abstract

**Background:**

There is no report of the application of intraoperative computed tomography to the extremities, and its usefulness is not mentioned.

**Case presentation:**

We present a case of a patient with the elbow pain and loss of the forearm rotation due to the prominent bicipital tuberosity of the radius, which was diagnosed as enthesopathy. Surgical treatment to excise the prominent part of the bicipital tuberosity of the radius was recommended. However, it is difficult to perform the appropriate excision of the abnormal prominent part because of complications such as bicipital tendon rupture. The patient was successfully treated by surgical resection under the control of intraoperative computed tomography.

**Conclusions:**

Intraoperative computed tomography scan is a useful tool to assess the remaining volume of the abnormal bones.

## Introduction

Enthesopathy of the bicipital tuberosity of the radius has been reported to be the enthesis of the insertion site of the bicipital tendon into the radius which causes limitation of the range of forearm rotation motion [1]. Resection of the tuberosity to improve the impingement between the tuberosity and the ulna is the treatment. It is, however, difficult to determine the volume of bone to resect before encountering complications such as bicipital tendon rupture. We report a case of bicipital tuberosity enthesopathy of the radius treated by surgical resection using an intraoperative computed tomography (CT) scan.

## Case report

**Fig. 1:**
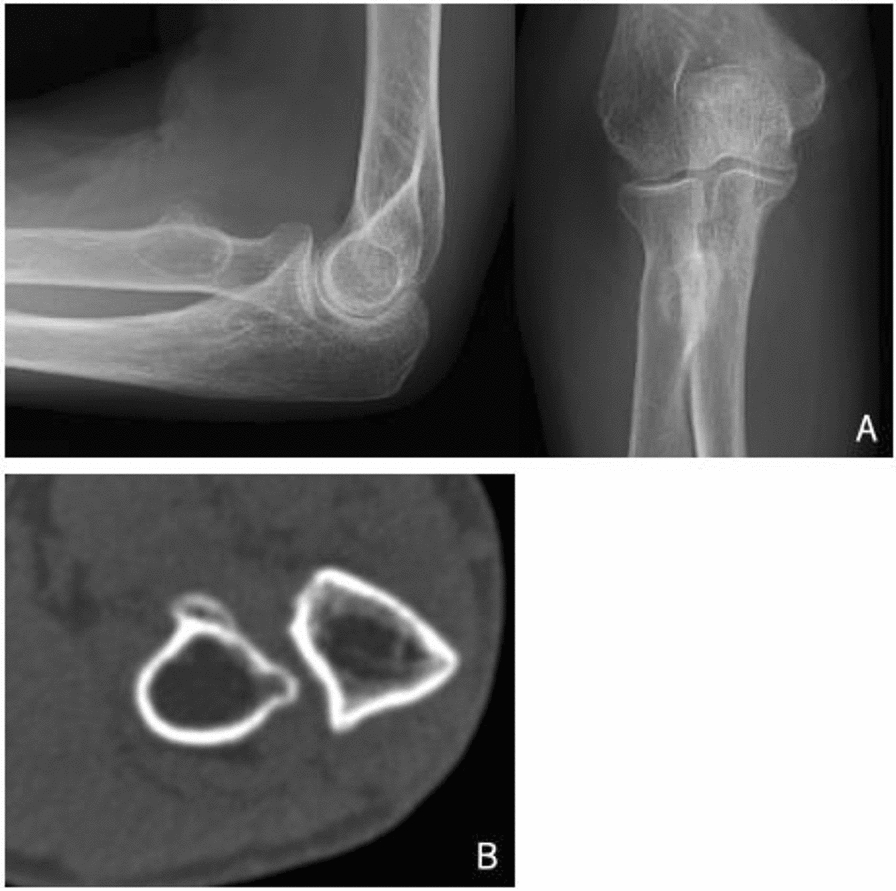
**A** Lateral and anteroposterior radiograph of the right elbow showed an abnormal prominence of the bicipital tuberosity of the radius. **B** Computed tomography of the right elbow showed abnormal spur formation of the bicipital tuberosity of the radius and the cortex of the ulna

A 77-year-old, right-handed female was referred to us with right elbow pain that began a few years ago. She had a restricted range of motion with 60° of supination and 30° of pronation with painful crepitus of her right elbow during forearm rotation. She had a full range of motion, with 145° of flexion and 0° of extension. Tenderness existed on the tuberosity of the radius without redness, swelling, muscle atrophy, or neurological deficits. Blood test results were within normal limits. She had no history of trauma or disease. Radiographs of the elbow showed an irregular prominence of the bicipital tuberosity of the radius (Fig. 1). CT scan also showed this irregular prominence of the bicipital tuberosity of the radius, as well as narrowing between the bicipital tuberosity and the ulna.

**Fig. 2:**
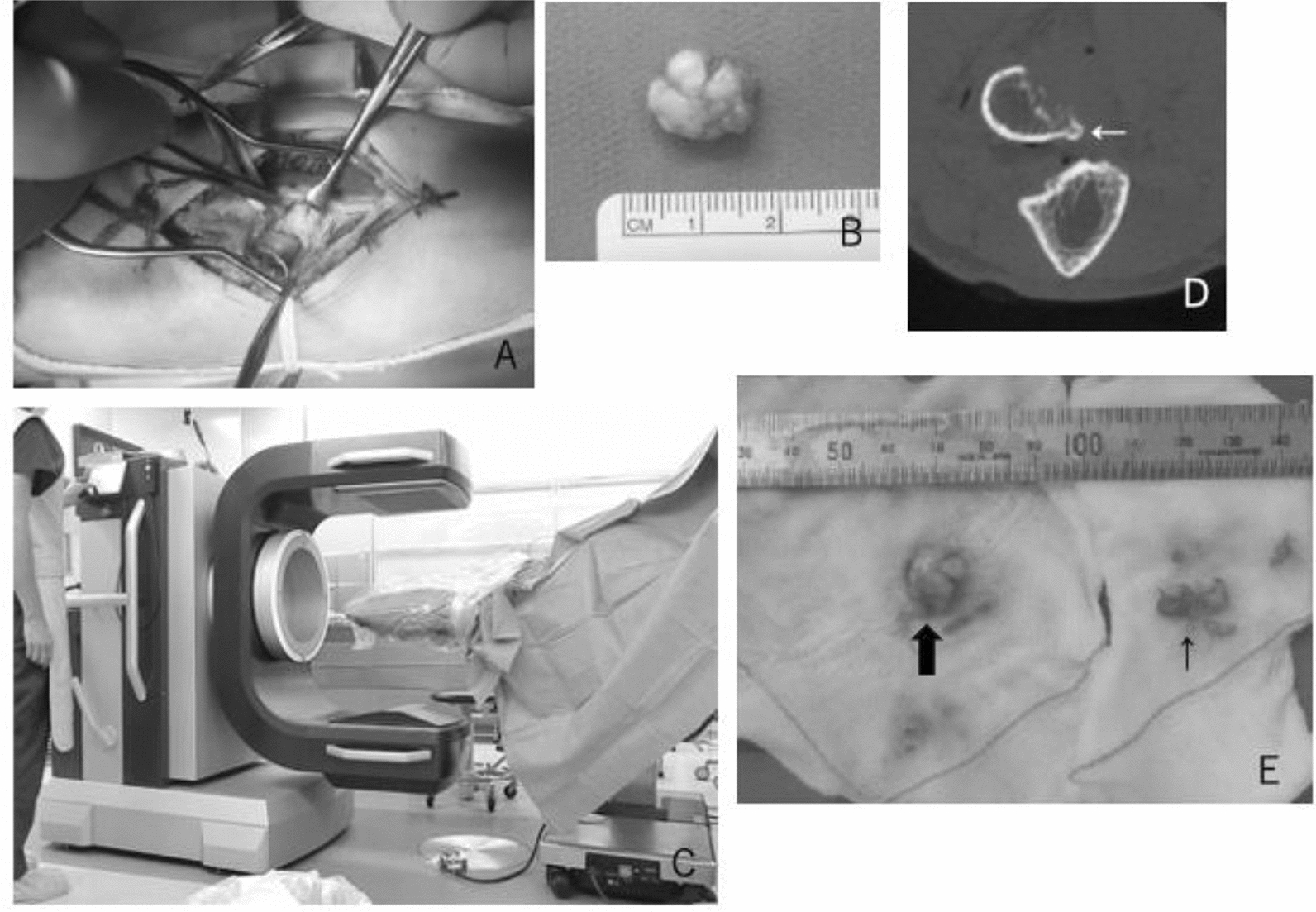
**A** Intraoperative picture of the left elbow showed the abnormal prominence of the bicipital tuberosity of the radius. **B** 11 mm × 15 mm of the lesion was removed. **C** Intraoperative CT scan was performed using 3D Accuitomo M (Morita Co. Ltd., Kyoto, Japan). **D** Intraoperative computed tomography of the right elbow showed the remaining spur of the bicipital tuberosity of the radius (arrow). **E** Additional resection was performed (small arrow). The large arrow shows the resected part before the computed tomography (already shown in **B**)

First, conservative treatment was tried, with daily activity instruction and oral medication nonsteroidal anti-inflammatory drugs for 3 months. The conservative treatment was not effective and the patient desired the operation. A skin incision was made on the anterior site of the proximal forearm. The bicipital tuberosity of the radius was exposed between the ulnar side of the brachioradialis muscle and the radial side of the biceps tendon (Fig. 2). The irregular prominence was excised from the base of the bicipital tuberosity. Intraoperative CT scan was performed using 3D Accuitomo M (Morita Co. Ltd., Kyoto, Japan), and the shape of the bicipital tuberosity was confirmed. Because the resected volume was not large enough to decrease the prominence, we also excised the remaining part of the irregular prominence. We confirmed the improvement of the range of motion of supination and pronation. After the irrigation, the superficial fascia and skin was sutured. Postoperatively, immobilization with a long arm cast was undertaken for 2 weeks. Sutures were removed 2 weeks after the operation. After removal of the cast, gentle active range of motion was started. The histological examination revealed normal bones with surrounding fibrous tissues, consistent with osteophyte.

**Fig. 3:**
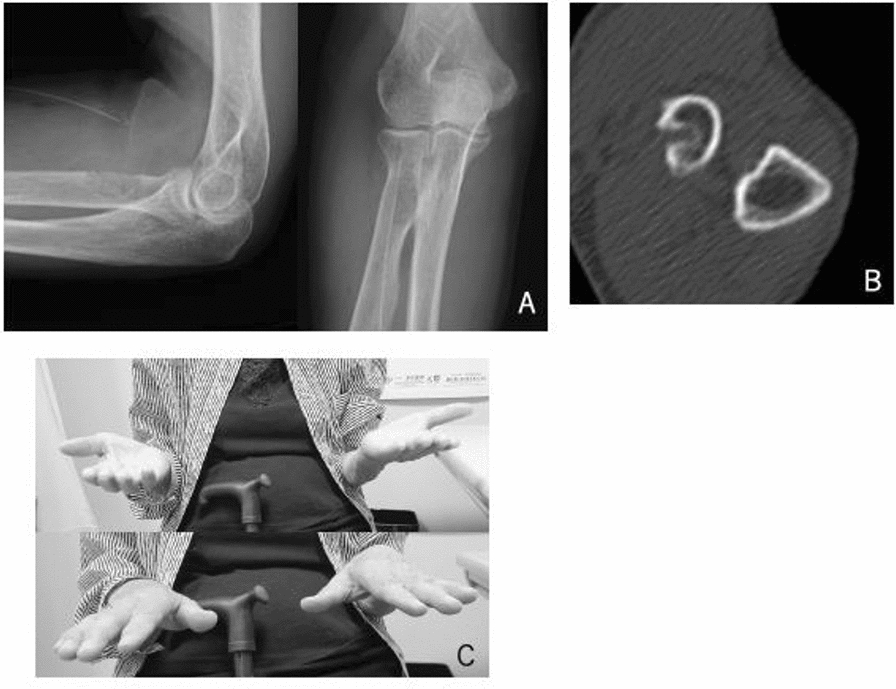
**A** Postoperative radiographs of the left elbow showed no abnormal prominence of the bicipital tuberosity of the radius. **B** Postoperative computed tomography of the left elbow showed no abnormal spur of the bicipital tuberosity of the radius. **C** The patient’s limitation of the forearm rotation of the left elbow improved

At the latest follow-up 1 year after the operation, the patient had no pain and no motor weakness or limitation in forearm rotation (Fig. 3). CT scan showed no abnormal prominence of the bicipital tuberosity of the radius.

## Discussion

Enthesopathy of the bicipital tuberosity of the radius has previously been reported [1–4]. Patient symptoms are elbow pain, limitation of the range of forearm rotation, and clicking during forearm rotation. These symptoms arise from the impingement between the prominence of the bicipital tuberosity and the ulnar cortex. Radiographic change shows spur formation and an irregular margin of the tuberosity of the radius, and sometimes spur formation of the ulnar cortex. CT scan reveals this bone change. If conservative treatment is not effective at improving patient symptoms and range of forearm rotation, surgical treatment to excise the prominent part of the bicipital tuberosity of the radius is recommended. To perform the appropriate excision of the abnormal prominent part, the correct anatomical relation of the biceps tendon and radial tuberosity is important. The distal biceps tendon inserts on the ulnar side of the tuberosity. The radial side of the tuberosity is roughened and covered by an overlying bursa, present on the radial side of the tendon. The insertion footprint does not cover the ridge and radial side of the tuberosity [2]. To prevent the detachment of the bicipital tendon, the surgical approach for excision of the prominence should be performed from the radial side of the biceps tendon and the bicipital tuberosity.

It is sometimes difficult to recognize which part of the tuberosity is to be resected because of the deformity of the irregular prominence of the bicipital tuberosity. To prevent the detachment of the bicipital tendon, resection is sometimes insufficient. In the current case, the intraoperative CT scan showed that the resection was insufficient to flatten the prominence and prevent impingement between the radius and the ulna. After assessment of the volume of remaining bone, we resected the abnormal prominence. At the postoperative follow-up, the patient’s pain and forearm rotation range of motion had improved. Intraoperative CT scan is a useful tool to assess the remaining volume and shape of abnormal bones during a bone resection. However, adequate identification of abnormal bones with a proper surgical exposure is easy for a skilled surgeon who can identify them without intraoperative CT scan. Intraoperative CT scan is not widely used, and current intraoperative fluoroscopy in proper position is also helpful when the surgical exposure is difficult.

In conclusion, enthesopathy of the bicipital tuberosity of the radius can be successfully treated by surgical resection, in which intraoperative CT scan is a useful tool to assess the remaining volume of the abnormal bones.

## Data Availability

The datasets collected and analyzed during the current study are available from the corresponding author upon reasonable request.
